# Supplementary material for: High-fidelity collisional quantum gates with fermionic atoms
Source: Nature. 2026 Apr 8;652(8110):602–8. doi: 10.1038/s41586-026-10356-3 (PMC13083236; doi:10.1038/s41586-026-10356-3)
Supplement: Supplementary file 1 — Supplementary Information, including Supplementary Figs. 1–4. [file 41586_2026_10356_MOESM1_ESM.pdf]

nature portfolio
https://doi.org/10.1038/s41586-026-10356-3

Supplementary information

High-fidelity collisional quantum gates with fermionic atoms

In the format provided by the authors and unedited

Supplementary information for:
High-fidelity collisional quantum gates with fermionic atoms

Petar Bojović,[1,2] Timon Hilker,[1,2,3] Si Wang,[1,2] Johannes Obermeyer,[1,2] Marnix Barendregt,[1,2] Dorothee Tell,[1,2] Thomas Chalopin,[1,2,4] Philipp M. Preiss,[1,2] Immanuel Bloch,[1,2,5] and Titus Franz[1,2]

[1] *Max-Planck-Institut für Quantenoptik, 85748 Garching, Germany*
[2] *Munich Center for Quantum Science and Technology, 80799 Munich, Germany*
[3] *Department of Physics and SUPA, University of Strathclyde, Glasgow, G4 0NG, United Kingdom*
[4] *Laboratoire Charles Fabry, Institut d'Optique Graduate School, CNRS, Université Paris-Saclay, 91127 Palaiseau, France*
[5] *Fakultät für Physik, Ludwig-Maximilians-Universität, 80799 Munich, Germany*

Fermi-Hubbard double-well analytical derivation

The Fermi-Hubbard Hamiltonian

$$\hat{H}_{\mathrm{FH}} = -t \sum_{\sigma \in \{\uparrow,\downarrow\}} \left(\hat{c}^{\dagger}_{\mathrm{L},\sigma} \hat{c}_{\mathrm{R},\sigma} + \mathrm{h.c.} \right) + U \sum_{i \in \{\mathrm{L},\mathrm{R}\}} \hat{n}_{i,\uparrow} \hat{n}_{i,\downarrow} \tag{S1}$$

for a double-well with two particles of opposite spin can be expressed in matrix form in the basis

$$\begin{cases} |\psi_1\rangle = |\uparrow,\downarrow\rangle = \hat{c}^{\dagger}_{L,\uparrow}\hat{c}^{\dagger}_{R,\downarrow}|0\rangle \\ |\psi_2\rangle = |\downarrow,\uparrow\rangle = \hat{c}^{\dagger}_{L,\downarrow}\hat{c}^{\dagger}_{R,\uparrow}|0\rangle = -\hat{c}^{\dagger}_{R,\uparrow}\hat{c}^{\dagger}_{L,\downarrow}|0\rangle \\ |\psi_3\rangle = |\uparrow\downarrow,0\rangle = \hat{c}^{\dagger}_{L,\uparrow}\hat{c}^{\dagger}_{L,\downarrow}|0\rangle \\ |\psi_4\rangle = |0,\uparrow\downarrow\rangle = \hat{c}^{\dagger}_{R,\uparrow}\hat{c}^{\dagger}_{R,\downarrow}|0\rangle \end{cases} \tag{S2}$$

as

$$\hat{H}^{\mathrm{dw},S_z=0}_{\mathrm{FH}} = \begin{bmatrix} 0 & 0 & -t & -t \\ 0 & 0 & t & t \\ -t & t & U & 0 \\ -t & t & 0 & U \end{bmatrix}. \tag{S3}$$

This matrix can be diagonalized, yielding the eigenvalues

$$\lambda_1 = 0, \quad \lambda_2 = U, \quad \lambda_3 = -J, \quad \lambda_4 = U + J \tag{S4}$$

and eigenvectors

$$\mathbf{e}_1 = \frac{1}{\sqrt{2}}\begin{bmatrix} 1 \\ 1 \\ 0 \\ 0 \end{bmatrix}, \qquad \mathbf{e}_2 = \frac{1}{\sqrt{2}}\begin{bmatrix} 0 \\ 0 \\ -1 \\ 1 \end{bmatrix},$$

$$\mathbf{e}_3 = \frac{1}{2\sqrt{U_{\mathrm{eff}}}}\begin{bmatrix} \sqrt{U_{\mathrm{eff}}+U} \\ -\sqrt{U_{\mathrm{eff}}+U} \\ \sqrt{U_{\mathrm{eff}}-U} \\ \sqrt{U_{\mathrm{eff}}-U} \end{bmatrix} \overset{t/U\to 0}{\to} \frac{1}{\sqrt{2}}\begin{bmatrix} 1 \\ -1 \\ 0 \\ 0 \end{bmatrix},$$

$$\mathbf{e}_4 = \frac{1}{2\sqrt{U_{\mathrm{eff}}}}\begin{bmatrix} -\sqrt{U_{\mathrm{eff}}-U} \\ \sqrt{U_{\mathrm{eff}}-U} \\ \sqrt{U_{\mathrm{eff}}+U} \\ \sqrt{U_{\mathrm{eff}}+U} \end{bmatrix} \overset{t/U\to 0}{\to} \frac{1}{\sqrt{2}}\begin{bmatrix} 0 \\ 0 \\ 1 \\ 1 \end{bmatrix},$$

where we have introduced the effective interaction

$$U_{\mathrm{eff}} = \sqrt{U^2 + 16t^2} \tag{S5}$$

and generalized spin-exchange

$$J = {}^1\!/_2 (U_{\mathrm{eff}} - U). \tag{S6}$$

Expanding this in small $\epsilon = t/U$ gives $U_{\mathrm{eff}} = U + 2\tilde{J} + \mathcal{O}(\epsilon^4)$ and $J = \tilde{J} + \mathcal{O}(\epsilon^4)$ with $\tilde{J} = 4t^2/U$.

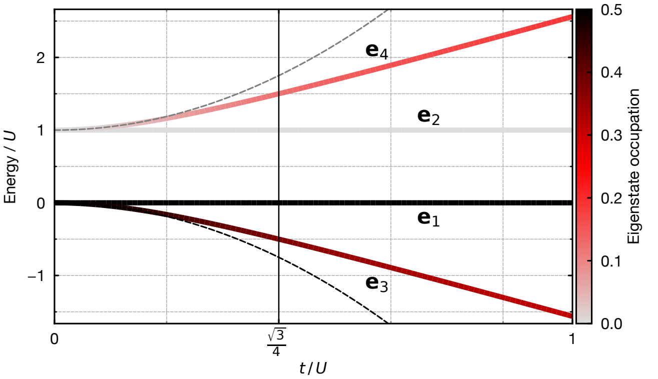

Figure S1. **Eigenvalues of the Fermi-Hubbard Hamiltonian**: Eigenvalues λ_i as a function of t/U for the four eigenvectors $\mathbf{e}_i$ in a double-well with two particles and total spin projection $S_z = 0$. The colorbar denotes the eigenstate occupation of $|\langle\uparrow,\downarrow|\mathbf{e_i}\rangle|^2$ for all four eigenstates $|\mathbf{e_i}\rangle$. The dashed lines represent the approximations to λ_3 and λ_4 in the limit $t/U \ll 1$. Marked as a vertical line is the magic ratio $t/U = \sqrt{3}/4$.

It helps intuition to analyze the eigensystem (see Fig. S1) in the limit of small hopping-to-interaction ratio ($t/U \ll 1$), where the eigenstates naturally separate into spin- and charge-dominated sectors. The lowest two eigenstates, $\mathbf{e}_1$ and $\mathbf{e}_3$, are predominantly spin-like (eigenstate occupations $|\langle\uparrow,\downarrow|\mathbf{e_i}\rangle|^2 \approx |\langle\downarrow,\uparrow|\mathbf{e_i}\rangle|^2 \approx \frac{1}{2}$, see also colorbar in Fig. S1) and are adiabatically connected as $t \to 0$. The state $\mathbf{e}_1$ corresponds to a spin-triplet configuration without any doublon occupation, while $\mathbf{e}_3$ exhibits only a small admixture of doubly occupied states. Consequently, the spin dynamics are primarily governed by the coherent evolution between these two states and occur at a characteristic energy scale J, given by their energy splitting. In contrast, the states $\mathbf{e}_2$ and $\mathbf{e}_4$ are charge-dominated (eigenstate occupations $|\langle\uparrow,\downarrow|\mathbf{e_i}\rangle|^2 \approx |\langle\downarrow,\uparrow|\mathbf{e_i}\rangle|^2 \approx 0$, see also colorbar in Fig. S1), composed mainly of configurations with one site doubly occupied and the other empty ($|\uparrow\downarrow,0\rangle$ and $|0,\uparrow\downarrow\rangle$). These charge excitations also exhibit an energy separation on the order of J, but are energetically offset from the spin sector by approximately U, the on-site interaction energy. This separation defines a clear hierarchy of

energy scales, allowing the spin and charge dynamics to be treated independently at leading order.

A natural protocol for initiating coherent dynamics is to prepare the system in a state $|\psi_m\rangle$ and perform a sudden quench of the tunneling amplitude from zero to a finite value $t > 0$. Following the quench, each eigenstate $\mathbf{e}_i$ acquires a dynamical phase, resulting in the time evolved state (with $\hbar = h/(2\pi)$)

$$\langle\psi_m|e^{-i\hat{H}_{\mathrm{FH}}\tau_{\mathrm{h}}/\hbar}|\psi_n\rangle = \sum_i \langle\psi_m|\mathbf{e}_i\rangle \langle\mathbf{e}_i|\psi_n\rangle\, e^{-i\lambda_i\tau_{\mathrm{h}}/\hbar}. \tag{S7}$$

It is instructive to visualize this time evolution as a sum of rotating vectors in the complex plane. Each vector corresponds to an eigenstate $\mathbf{e}_i$, with its amplitude given by the product of wavefunction overlaps $|\langle\psi_m|\mathbf{e}_i\rangle\langle\mathbf{e}_i|\psi_n\rangle|$, and its phase evolving at frequency λ_i.

As a concrete example, consider the matrix element $\langle\uparrow,\downarrow|e^{-i\hat{H}_{\mathrm{FH}}\tau_{\mathrm{h}}/\hbar}|\uparrow,\downarrow\rangle$. This evolution is determined by three vector contributions (see Fig. S2). The first, associated with eigenstate $\mathbf{e}_1$, has an overlap amplitude of $\frac{1}{2}$ and an eigenvalue $\lambda_1 = 0$, meaning this vector remains stationary over time. For $t/U \ll 1$, the second most significant contribution arises from eigenstate $\mathbf{e}_3$, rotating at frequency J. This term drives the spin-exchange dynamics. The overlap of $|\uparrow,\downarrow\rangle$ with $\mathbf{e}_2$ is exactly zero, and with $\mathbf{e}_4$ is zero in the limit of $t/U \ll 1$. In summary, evaluating equation (S7) in this limit, leads to

$$\begin{aligned}
&\langle\uparrow,\downarrow|e^{-i\hat{H}_{\mathrm{FH}}\tau_{\mathrm{h}}/\hbar}|\uparrow,\downarrow\rangle \\
&\overset{t/U\ll 1}{\approx} \left|\langle\uparrow,\downarrow|\mathbf{e}_1\rangle\right|^2 e^{-i\lambda_1\tau_{\mathrm{h}}/\hbar} + \left|\langle\uparrow,\downarrow|\mathbf{e}_3\rangle\right|^2 e^{-i\lambda_3\tau_{\mathrm{h}}/\hbar} \\
&\overset{t/U\ll 1}{\approx} \frac{1}{2} + \frac{1}{2}e^{iJ\tau_{\mathrm{h}}/\hbar}.
\end{aligned} \tag{S8}$$

Similarly, the complete time evolution operator can be derived as:

$$U_{\mathrm{int}}(\theta = J\tau_{\mathrm{h}}/\hbar) \overset{t/U\ll 1}{=} \begin{bmatrix} \frac{1+e^{iJ\tau_{\mathrm{h}}/\hbar}}{2} & \frac{1-e^{iJ\tau_{\mathrm{h}}/\hbar}}{2} & 0 & 0 \\ \frac{1-e^{iJ\tau_{\mathrm{h}}/\hbar}}{2} & \frac{1+e^{iJ\tau_{\mathrm{h}}/\hbar}}{2} & 0 & 0 \\ 0 & 0 & \mathrm{e}^{-iU\tau_{\mathrm{h}}/\hbar}\,\frac{1+e^{-iJ\tau_{\mathrm{h}}/\hbar}}{2} & -\mathrm{e}^{-iU\tau_{\mathrm{h}}/\hbar}\,\frac{1-e^{-iJ\tau_{\mathrm{h}}/\hbar}}{2} \\ 0 & 0 & -\mathrm{e}^{-iU\tau_{\mathrm{h}}/\hbar}\,\frac{1-e^{-iJ\tau_{\mathrm{h}}/\hbar}}{2} & \mathrm{e}^{-iU\tau_{\mathrm{h}}/\hbar}\,\frac{1+e^{-iJ\tau_{\mathrm{h}}/\hbar}}{2} \end{bmatrix}, \tag{S9}$$

Which is exactly the lower 4x4 block of the interaction matrix $U_{\mathrm{int}}(\theta)$ from equation (2) with $\theta = J\tau_{\mathrm{h}}/\hbar$ and $\zeta = U\tau_{\mathrm{h}}/\hbar$. Note that the Fermi-Hubbard Hamiltonian (S1) preserves the projection of the total spin $\hat{S}_z$, therefore it does not couple the states $|\uparrow,\uparrow\rangle$ and $|\downarrow,\downarrow\rangle$ to any other state. The exchange process described in our work couples two states that do not directly interact via a virtual intermediate state that is energetically detuned. This results in a second-order, effectively coherent two-body interaction.

Errors to the SWAP$^\alpha$ gate arise firstly from a small contribution from eigenstate $\mathbf{e}_4$ whose vector rotates rapidly with frequency $U + J$ (see gray vector in the bottom row in Fig. S2). This high-frequency component introduces a phase and amplitude error that scales as $U_{\mathrm{eff}} - U \approx 2\tilde{J} + U\mathcal{O}(\epsilon^4)$. In addition, for the initial state $|\uparrow,\downarrow\rangle$, the doublon population $|\langle\uparrow\downarrow,0|\uparrow,\downarrow\rangle|^2$ oscillates coherently with frequency U_{eff} and peak-to-peak amplitude $4t^2/U_{\mathrm{eff}}^2$ (see orange line in Fig. S2):

$$\begin{aligned}
&\langle\uparrow\downarrow,0|e^{-i\hat{H}_{\mathrm{FH}}\tau_{\mathrm{h}}/\hbar}|\uparrow,\downarrow\rangle \\
&\quad= \langle\uparrow\downarrow,0|\mathbf{e}_3\rangle\langle\mathbf{e}_3|\uparrow,\downarrow\rangle\, e^{-i\lambda_3\tau_{\mathrm{h}}/\hbar} \\
&\qquad+ \langle\uparrow\downarrow,0|\mathbf{e}_4\rangle\langle\mathbf{e}_4|\uparrow,\downarrow\rangle\, e^{-i\lambda_4\tau_{\mathrm{h}}/\hbar} \\
&\quad= \frac{t}{U_{\mathrm{eff}}} e^{-\frac{i}{2}(U-U_{\mathrm{eff}})\tau_{\mathrm{h}}/\hbar} - \frac{t}{U_{\mathrm{eff}}} e^{-\frac{i}{2}(U+U_{\mathrm{eff}})\tau_{\mathrm{h}}/\hbar} \\
&\quad= \frac{2i\,t}{U_{\mathrm{eff}}}\, e^{-\frac{iU\tau_{\mathrm{h}}}{2\hbar}} \sin\left(\frac{U_{\mathrm{eff}}\tau_{\mathrm{h}}}{2\hbar}\right)
\end{aligned} \tag{S10}$$

This equation holds generally for arbitrary U/t because $U_{\mathrm{eff}} = \lambda_4 - \lambda_3$ is the energy difference of the two populated eigenstates with doublon-hole population.

To speed up the gates, the ratio U/t can be reduced, however this increases the influence of the fast-rotating $\mathbf{e}_4$ vector, leading to a substantial phase and amplitude error of the SWAP$^\alpha$ gate unless $U_{\mathrm{eff}} \cdot \tau_{\mathrm{h}}$ is an integer multiple of 2π [Ref. 13 in the main text]. This leads to a series of *magic ratios* for U/t. For an entangling $\pi/2$ pulse, the gate time is $\tau_{\mathrm{h}} = \hbar\pi/(2J)$ leading to the condition

$$\begin{aligned}
U_{\mathrm{eff}} \cdot \tau_{\mathrm{h}}/\hbar &= \frac{U_{\mathrm{eff}}\,\pi}{2J} \overset{!}{=} 2\pi n \\
\Rightarrow \left(\frac{U}{t}\right)_{\text{magic } \pi/2} &= \frac{4(2n-1)}{\sqrt{4n-1}},
\end{aligned}$$

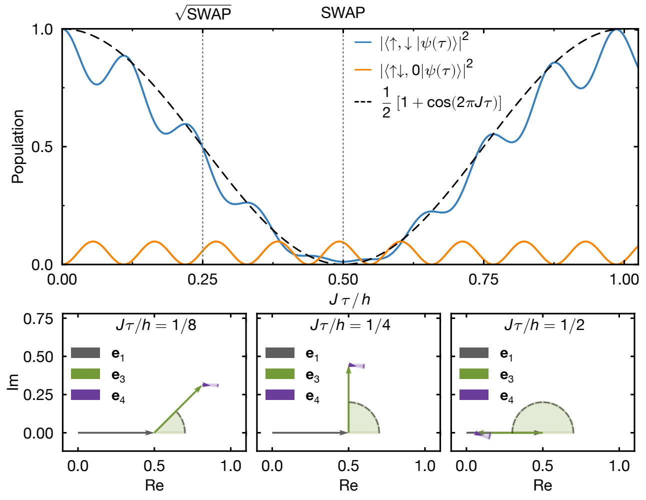

Figure S2. **Time evolution of $|\psi_0\rangle = |\uparrow,\downarrow\rangle$ for $U/t = 5$ and for a quench of the lattice depth**: Top: Time evolution of the population in $|\uparrow,\downarrow\rangle$ (blue) and in the doublon state $|\uparrow\downarrow,0\rangle$ (orange). The dashed black line shows an oscillation at frequency J. Lower panels: Visualization of the time evolution as a sum of rotating vectors in the complex plane. Each vector represents an eigenstate $\mathbf{e}_i$, with an amplitude given by the product of wavefunction overlaps $|\langle\psi_m|\mathbf{e}_i\rangle\langle\mathbf{e}_i|\psi_n\rangle|$ and a phase that evolves at the eigenfrequency λ_i.

where the integer n gives the number of full 2π rotations of the doublon-hole population during the time of the $\pi/2$ exchange gate. Explicitly, $\Rightarrow \left(\frac{U}{t}\right)_{\text{magic }\pi/2} \in \{4/\sqrt{3},\ 12/\sqrt{7},\ 20/\sqrt{11}...\}$, enable the realization of maximally entangled Bell states. For arbitrary $\alpha = \theta/\pi$ the *magic ratio* pulse is generalized to:

$$\left(\frac{U}{t}\right)_{\pi\alpha} = \frac{4(n-\alpha)}{\sqrt{\alpha(2n-\alpha)}},$$

The magic ratio with the fastest dynamics, and thus the fastest gate operation of this protocol, is $U/t = 4/\sqrt{3}$, for which the effective interaction is $U_{\text{eff}} = 2U = 8t/\sqrt{3}$, and spin-exchange is $J = U/2 = 2t/\sqrt{3}$. This quantifies how the collisional gate speed is ultimately set by the interaction strength. Evaluating all matrix elements in equation (S7) for $U/t = 4/\sqrt{3}$ and $\tau_{\text{h}} = h/(4J)$ again yields the interaction matrix

$$U_{\text{int}} \overset{\frac{U}{t}=\frac{4}{\sqrt{3}}}{\underset{\tau_{\text{h}}=\frac{h}{4J}}{=}} \begin{pmatrix} \frac{1}{2}+\frac{i}{2} & \frac{1}{2}-\frac{i}{2} & 0 & 0 \\ \frac{1}{2}-\frac{i}{2} & \frac{1}{2}+\frac{i}{2} & 0 & 0 \\ 0 & 0 & -\frac{1}{2}+\frac{i}{2} & \frac{1}{2}+\frac{i}{2} \\ 0 & 0 & \frac{1}{2}+\frac{i}{2} & -\frac{1}{2}+\frac{i}{2} \end{pmatrix}$$

which is again exactly the lower 4x4 diagonal block of $U_{\text{int}}(\theta)$ with $\theta = J\tau_{\text{h}}/\hbar = \frac{\pi}{2}$ and $\zeta = U\tau_{\text{h}}/\hbar = \pi$. The same analysis as in the previous $U/t \gg 1$ is shown in Fig. S3. In theory, this condition provides an optimal realization of the $\sqrt{\text{SWAP}}$ gate. However, in practice, it

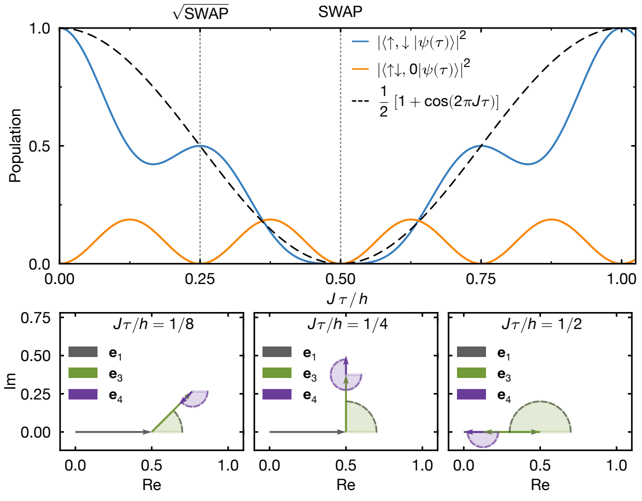

Figure S3. **Time evolution of $|\psi_0\rangle = |\uparrow,\downarrow\rangle$ for the magic ratio** $U/t = 4/\sqrt{3}$: Otherwise same as Fig. S2.

is experimentally demanding to fine-tune both the interaction ratio U/t and the pulse duration with the required precision.

An alternative approach to implementing the gate is to ramp the tunneling amplitude t quasi-adiabatically from zero to its final value. Provided the ramp is slow compared to the interaction scale U, the system evolves within the subspace spanned by the eigenstates $\mathbf{e}_1$ and $\mathbf{e}_3$, which remain predominantly populated. Consequently, the amplitude of the vector associated to $\mathbf{e}_4$ remains zero throughout the evolution. This eliminates amplitude and phase errors, as well as any unwanted coupling between the spin and charge sectors. Importantly, there is no requirement for a minimal gate speed: since $\mathbf{e}_1$ and $\mathbf{e}_3$ are degenerate at $t = 0$ the overlap with these two states is always exactly equal. Additionally, the specific shape of the pulse is inconsequential, provided it remains sufficiently slow. The resulting gate angle α is determined solely by the total duration of the pulse τ_{total} and the time-dependent exchange interaction $J(\tau_{\text{p}})$, according to:

$$\alpha = \frac{1}{\pi}\int_0^{\tau_{\text{total}}} J(\tau_{\text{p}})/\hbar \, d\tau_{\text{p}}. \qquad \text{(S11)}$$

Fig. S4 shows the evolution of the populations during Blackman pulses of different length demonstrating the suppression of doublon-hole population.

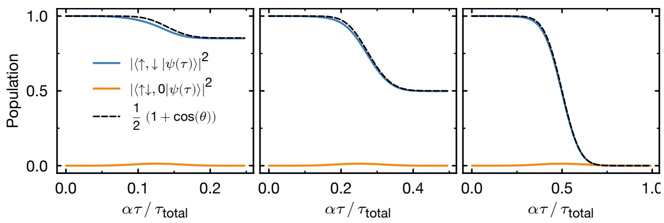

Figure S4. **Blackman Pulses**: Time evolution of the population in $|\uparrow,\downarrow\rangle$ (blue solid line) and $|\uparrow\downarrow,0\rangle$ (orange solid line) for quasi-adiabatic pulses with $\alpha \in \{\frac{1}{4}, \frac{1}{2}, 1\}$ starting in $|\uparrow,\downarrow\rangle$. For all pulses, the tunneling amplitude t is ramped according to a Blackman profile, while the on-site interaction U remains constant, with ratio of $U/t > 8$. For all three resulting gate angles, the final state has negligible doublon population, and the population in $|\uparrow,\downarrow\rangle$ is well described by equation (S11) (black dashed line).
